# Clinical incident reporting behaviors and associated factors among health professionals in Dessie comprehensive specialized hospital, Amhara Region, Ethiopia: a mixed method study

**DOI:** 10.1186/s12913-021-07350-y

**Published:** 2021-12-11

**Authors:** Zemen Mengesha Yalew, Yibeltal Asmamaw Yitayew

**Affiliations:** 1 Department of Comprehensive Nursing, College of Medicine and Health Science, Wollo University, Dessie, Ethiopia; 2 Department of Paediatrics Nursing, College of Medicine and Health Science, Wollo University, Dessie, Ethiopia

**Keywords:** Ethiopia, Health professional, Clinical Incident reporting

## Abstract

**Background:**

Understanding the type and causes of errors are necessary for the prevention of occurrence or reoccurrence. Therefore addressing the behavior of health professionals on reporting clinical incidents is crucial to create spontaneous knowledge from mistakes and enhance patient safety.

**Method:**

A mixed type institution-based cross-sectional study design was conducted from March 1 - 30, 2020 in Dessie comprehensive specialized hospital among 319 and 18 participants for the quantitative and qualitative study, respectively. The professions and participants with their assigned proportions were selected using a simple random sampling technique. For quantitative and qualitative data, semi structured questionnaires and interviewer-guided questions were used to collect data, respectively. Finally, qualitative findings were used to supplement the quantitative result.

**Result:**

The finding showed that the proportion of clinical incident reporting behavior among health professionals was 12.4%. Having training (AOR=3.6, 95% CI, 1.15-11.45), incident reporting help to minimize errors (AOR=2.8, 95% CI, 1.29-6.02), fear of legal penalty (AOR= 0.3, 95% CI, 0.13-0.82), and lack of feedback (AOR=0.3, 95% CI, 0.11-0.90) were identified as significant factors for clinical incident reporting behavior of the health professionals.

**Conclusions:**

This study showed that the clinical incident reporting behavior of the health professionals was very low. Therefore health professionals should get training on clinical incident reporting and the hospital should have an incident reporting system and guideline.

**Supplementary Information:**

The online version contains supplementary material available at 10.1186/s12913-021-07350-y.

## Introduction

Patient safety has attention internationally due to the rise of awareness on the occurrences of medical errors [1]. Patient safety culture is an important aspect of quality healthcare delivery and is an issue of high concern globally [2]. Medical errors are a cause of mortality and sickness all throughout the world, and they continue to be the main cause of morbidity and mortality [3, 4].

A medical error is defined as an injury caused by medical therapy rather than the patient’s underlining condition. An unintentional unpleasant event can be avoided. A variety of factors can trigger almost all unfavorable events. They may be unsafe acts committed by people who are in direct contact with the patient or system [5, 6]. Adverse drug events and improper transfusions, misdiagnosis, under, and overtreatment, surgical injuries and wrong-site surgery, suicides, restraint-related injuries or death, falls, burns, pressure ulcers, and misidentification of the patient are just a few of the problems that can arise in the course of providing health care [6]. Throughout the world, incident reporting systems have been established, some at the health care facility or organizational level, some at the national health system level [7]. In a health care facility, a comprehensive and systematic approach to incident reporting would help in the learning from errors and adverse events [8, 9]. According to studies, reporting systems detect 7–15% of adverse events [10], and health care facilities, in general, need to change policies and procedures that encourage health workers to disclose incidents. The policies also create the chance for health workers to learn from mistakes and this will also further enhance patient safety [11].

Clinical incident disclosing allow the healthcare system to be adjusted, and they give incredibly significant information for the prevention of errors by detecting systemic flaws that are still present and could expose the patient to medical adverse events. It can also be used to detect preventable events if it is practiced well within clinical settings, but many of the incidents are not disclosed or are simply not recognized due to not feel free to disclose errors, fear of punishment, blame, and shame [8, 12, 13].

In Ethiopia, patient safety culture is a relatively recent problem, and little is known about how it is implemented in public hospitals. Improving patient safety requires creating a culture that supports and encourages healthcare workers to disclose errors [14]. Addressing the behaviors of health workers on disclosing clinical incidents is important to create spontaneous knowledge from mistakes and this will also further provide valuable information for the prevention of future errors and enhance patient safety [11]. Therefore, this study was aimed to assess the clinical incident reporting behavior of the health worker in Dessie comprehensive specialized hospital, Amhara Ethiopia.

## Methods

### Study design, area and period

A mixed type institution-based cross-sectional study was conducted at Dessie comprehensive specialized hospital from March 1 to March 30, 2020, using a survey questionnaire and focus group discussion for quantitative and qualitative studies respectively. A mixed method study is a one-phased study that begins with the concurrent collection of quantitative and qualitative data. To explain and expand the results, the simultaneous linkage of quantitative and qualitative data was used. Dessie comprehensive specialized hospital is found in Dessie town which is the capital city of South Wollo Zone, Amhara region. The town is located 400 km from Addis Ababa, the capital city of Ethiopia, and 462 Km from Bahir Dar, the capital city of the Amhara region. The Hospital provides multidimensional aspects of care to clients who need health care services including; pediatrics, medical, surgical, gynecological, and other services.

### Population

#### Source population

All health professionals in Dessie comprehensive specialized hospital.

#### Study population

Randomly selected health professionals in Dessie comprehensive specialized hospital.

### Sample size determination and procedure

The sample size was determined using a single population proportion formula by considering a 25.4% proportion of incident reporting behavior of health professionals conducted in Gondar university comprehensive specialized hospital, Ethiopia [9], with a 95% CI and 5% marginal error. Considering 10% non-response rate the final sample size was 319. Professions (Nursing, Midwifery, Pharmacy, Medicine, and Anesthesia) and participats from each profession were selected using simple random sampling technique for quantitative study. Three focus group discussions with eighteen (18) discussants were undertaken for the qualitative study until the point in data collection when new insights into the research questions were no longer viable.

### Eligibility criteria

#### Exclusion criteria

Health professionals on annual leave were excluded.

### Data collection tool and procedure

Data were collected by using a structured self-administered questionnaire. The data collection tool was adapted from a study conducted in Gondar specialized referral hospital [9]. The tool comprises four parts. These are the Sociodemographic characteristics (age, sex, ethnicity, religion, profession, educational level, year of service, working hour per week, and work unit), institutional factors (training on clinical incident reporting behavior, availability of guideline/policy, and availability of reporting formats), perceived barriers on clinical incident reporting behavior (non-supportive environment (culture of shame and blame), loss of prestige among colleagues, fear of legal penalties, fear of administrative suction and lack of feedback), and clinical incident reporting behavior of the health professionals. In addition, the qualitative data were collected by using an unstructured interviewer guide through focused group discussion. Four Bachelor of Science (BSc) Nurses collected the data under the supervision of one Master of Science (MSc) Nurse.

### Variables

#### Dependent variable

 Clinical incident reporting behavior.

### Independent variable

**Sociodemographic characteristics**: age, sex, ethnicity, religion, department, educational level, year of service, working hour per week, and work unit.

**Institutional factors**:training on clinical incident reporting behavior, availability of guideline/policy, and availability of reporting formats.

**Perceived barriers:** non-supportive environment (culture of shame and blame), loss of prestige among colleagues, fear of legal penalties, fear of administrative suction and lack of feedback.

### Operational definition

#### Clinical Incident reporting behavior of health professionals

 Health professionals’ action towards the error they committed to disclose or conceal to their colleague or concerned body [9, 15].

**High clinical incident reporting behavior:** health professionals who choose the option “always” for all items [9].

**Low clinical incident reporting behavior:** health professionals who choose one of the options **“**most of the time”, “sometimes”, “rarely”, or “not disclose at all” for one or more item(s) [9].

### Data quality assurance

The Pre-test was conducted on 5% of the final sample size in Akasta general hospital one week before the actual data collection. The quality of data was ensured through training of data collectors, regular supervision, immediate feedback, and reviewing each of the questionnaires daily by the principal investigators. Double data entry was performed, and the consistency of the entered data was cross-checked. Field notes and audio records were checked for their appropriateness in terms of proper coding and clear audibility.

### Judgment criteria

To ensure the study’s validity, we considered four criterias: dependability, conformability, credibility, and transferability [16]. The triangulation of various components of data gathering ensured the data acquired in this study was trustworthy: (A) incorporating health professionals from different departments, (B) Researchers with relevant experience were invited to participate. All of the researchers were examined and the research findings were determined to be trustworthy.

### Data processing and analysis

The overall clinical incident reporting behavior of the health professionals was determined through the summation of clinical incident reporting behavior measuring items. For data entry and analysis, Epi data version 4.4.2 and Statistical Package for Social Sciences version 25 software were used. Descriptive statistics such as proportions, percentages, frequency distribution, and graphical presentation were used to describe the data. Variables with a p-value of less than and equal to 0.25 were entered into multivariable regression analysis and variables with p values < 0.05 were considered as statistically significant factors of clinical incident reporting behavior. For qualitative data, verbatim transcription in the Amharic language was made. The transcribed text file was translated into the English language for analysis. The translated text file was analyzed by using thematic content analysis. Finally, qualitative findings were used to supplement the quantitative result.

## Result

### Sociodemographic characteristics of the respondents

A total of 291 study participants have participated in the study with a 91% response rate. About 171(58.8%) of the respondents were male, 51.5% were married, 151(51.9%) of the respondents were orthodox, and 98.3% were Amhara in ethnicity. The median age of the respondent was 29 with (IQR=27-32), and 92.4% of respondents had no incident reporting training. 90% (90%) and 94.2% of respondents reported that the hospital did not have a reporting guideline and reporting format respectively (Table 1).

**Table 1:** Socio-demographic characteristics of the study participants on clinical incident reporting behavior in Dessie comprehensive specialized hospital, (n=291)

Variable	Category	Frequency	
		Number	Percent (%)
Age	<= 29	156	56.6
	30-39	103	35.4
	>=40	23	7.9
Sex	Male	171	58.8
	Female	120	41.2
Profession	Nursing	168	57.7
	Anesthesia	10	3.4
	Midwifery	29	10.0
	Medicine	64	22.0
	Pharmacy	20	6.9
Educational status	2nd degree and above	10	3.4
	1st degree	254	87.3
	Diploma	27	9.3
Working unit	Medicine	55	18.9
	Surgery	61	21.0
	Pediatrics	74	25.4
	Gynecology and obstetric	48	16.5
Work Experience	Outpatient department	14	4.8
	Emergency	24	8.2
	Other	15	5.2
	<=5	218	74.9
	6-10	51	17.5
	11-15	11	3.8
	>=16	11	3.8
Working hour /week	<=39	14	4.8
	40-59	162	55.7
	>=60	115	39.5

Other; OR: Operation room; ART: Antiretroviral treatment center

In a focus group discussion, there were 18 discussants. Five respondents were females, age of the respondents ranges from 21 to 40 years, and 7, 2, 4, 2, and 3 of respondents were nursing, medicine, midwifery, anesthesia and pharmacy respectively. The majority of respondents had a Bachelor of Science (BSc) degree.

After data analysis through reviewing the discussant’s ideas, three main themes and three subthemes were created. These were clinical incident reporting behavior, barriers in reporting errors, and suggestions for better clinical incident reporting practice. Lack of organizational structure, fear of the consequence, and lack of investigation on the cause of errors were subthemes under barriers in reporting errors.

### Clinical incident reporting behaviour of the participants

After the summation of the three Likert scale outcome variable measuring items, the proportion of health professionals reporting behavior of errors was 12.4% (95%CI, 8.93- 16.39).

In a qualitative study, nearly all respondents mentioned that there is no clinical incident reporting practice as well as a reporting system for medical errors in the health institution.

34 years male anaesthetist said that:

*“I don’t know about the reporting system on medical errors, but I hear mistakes when the patient or the patient’s family is sued. But I don’t know-how, and to whom incidents should be disclosed”* **(P4f)**.

35 years male midwife added:

*“…Medical errors are often heard from the patient or their family. There is no standard reporting system. In our unit, sometimes we discuss medical errors in the morning sessions for learning purpose”* **(P5t)**.

27 male pharmacist added more:

*“As they said, there is no formal reporting system; we also don’t know how and to whom incidents should be disclosed”* **(P6f).**

### Factors affecting clinical incident reporting behavior

In the bivariate logistic regression, respondents age, taking training, reporting errors help to minimize errors, fear of blame, fear of loss of prestige, legal penalty, and lack of feedback had a p-value <=0.25, and through the multivariable analysis, taking training, clinical incident reporting help to minimize errors, fear of penalty and lack of feedback were factors associated with clinical incident reporting through having p-value < 0.05 and 95% CI. Health professionals who had training on clinical incident reporting were 3.6 times more likely to disclose incidents than those who had no training (Adjusted odd ratio: (AOR)=3.6 (95% CI, 1.15,11.45)), those who perceive clinical incident reporting help to minimize errors were 2.8 times more likely to disclose incidents than those who did not perceive (AOR=2.8(95% CI, 1.29, 6.02)), those who fear legal penalty were 67.8% less likely to disclose incidents than those did not fear legal penalty (AOR=0.3(95% CI, 0.13, 0.82)), and lastly health worker who perceive lack of feedback was 69.3% less likely to disclose incidents than those who did not perceive so (AOR=0.3(95% CI, 0.11, 0.90)) ( Table 2).

**Table 2:** Multivariate analysis of factors associated with clinical incident reporting behavior among health professionals in Dessie Comprehensive Specialized Hospital, (n=291)

Variables	Category	Incident reporting		COR (95%CI)	AOR (95%CI)	P -value
		High	Low			
Age	<= 29	16	149		1	
	30-39	15	88	1.6(.75-3.37)	2.0(.92-4.63	.079
	>=40	5	18	2.6(.85-7.90)	2.9(.86-9.63)	.086
Taking training	Yes	6	16	2.9(1.09-8.22)	**3.6(1.15-11.45)**	**.027**
	No	30	239		1	
To minimize errors	Yes	18	76	2.4(1.16-4.77)	**2.8(1.29-6.02)**	**.009**
	No	18	179		1	
Fear of blame	Yes	11	109	.6(.28-1.25)	.4(0.20-1.06)	.069
	No	25	146		1	
Loss of prestige	Yes	3	46	.4(.12-1.40)	.3(.09-1.27)	.111
	No	33	209		1	
Fear of legal penalty	Yes	8	88	.5 (.24-1.24)	**.3(.13- .82)**	**.017**
	No	28	167		1	
Lack of feed back	Yes	5	69	.4(.16-1.16)	**.3(.11- .90)**	**.031**
	No	31	186		1	

AOR: adjusted odd ration; COR: crude odd ration

In the qualitative study, discussants also complained about some factors inhibiting reporting clinical errors. These factors were lack of organizational structure, fear of consequence, and lack of investigation on the cause of errors.

### Lack of Organizational Structure

The discussants mentioned that lack of organizational structure was a factor that affects reporting incidents.

For example, a 34 years male anesthetist said that:

*“…it may be related to the hospital management; there is no assigned responsible body to handle medical errors in the hospital”* (**P4f).**

32 years female pharmacist said that:

*“As mentioned earlier there is no reporting system and reporting format in our department”* **(P4s).**

Another 28 years female nurse added more:

“*As other members said, I do not know any reporting system, and also I have never seen a trained health professional and training on medical errors…”* (**P2f)**.

### Fear of consequence

Fear of the consequence that might occur after reporting errors. For example, a 29 years anesthetist said that:

“*As my friend said, the reporting system is weak…the main reason might be blaming from colleagues and managers”* **(P2t).**

Another 28 years male general practitioner added more:

“…*the reason for low incidents reporting practice might be fear of penalties and suctions”* (**P3f**).

### Lack of investigation on the causes of errors

Participants in the discussion said that a Lack of investigation on the cause of errors makes the health professionals not disclose errors.

A 21 years female midwife said that:

*“…errors are not investigated. To reduced incidents, there should be an investigation on the cause of errors”* **(Pt1)**.

Another 40 years nurse pointed out that:

*“…what keeps us from reporting medical errors is that; the cause of the errors is not investigated”* (p1f*).*

### Suggestions for having better reporting practice in the future

There were various suggestions raised by discussants for better clinical incident reporting practice in the future. For example, a 29 years male anesthetist said:

*“…there should be an incident officer in the hospital to receive and investigate all incidents discloses”* (P2t*).*

Another 40 years old male nurse added:

*“It is better to have clinical incident reporting guidelines, a format, and training…”* **(p1s)**.

## Discussion

The overall clinical incident reporting behavior of the health professionals in this finding was 12.4%. Correspondingly, the qualitative finding showed that nearly all respondents mentioned, there is no clinical incident reporting practice in the health institution, and also they did not know any reporting system on clinical incidents. This finding is lower than a study conducted in Egypt, Korea, Iran, and Ethiopia [8, 9, 17–20]. The discrepancy might be attributed to the inclusion of different disciplines, blaming in the working environment, lack of confidence in health workers, administrative punishment, and fear.

Participants who had training on clinical incident reporting had 3.6 times higher odds of reporting errors than those who had not been trained. This finding is in line with a study conducted in Gondar comprehensive specialized hospital [9]. Training on clinical incident reporting significantly improves the health worker’s skills in analyzing the factors of incidents with the prevention of future occurrence of medical errors. In addition, Training can improve systematic and structural strategies for recognizing, reporting, learning about, and characterizing incidents [21], and increasing the health care professional awareness on reporting medical errors can promote effectiveness to protect patients from incidents [22].

The present study showed that health workers who believe that reporting medical errors help to minimize errors were 2.8 times more likely to disclose incidents than those who did not believe so. Reporting medical errors is essential to improve patient safety and allow front-line health workers to learn concerning the causation of the incident, and systemic changes which will prevent it from recurring [23].

The other factor which affects the health workers’ clinical incident reporting behavior is fear of legal penalty. Health providers who fear legal penalties were 66.8% less likely to disclose medical errors than those who did not fear legal penalties. In the qualitative study, fear of the consequence that might occur after reporting errors makes the health care workers not disclose incidents. Fear of consequence was also identified by a study conducted in Iran [24]. A similar result revealed in Gondar’s comprehensive specialized hospital [9]. Literature supported that fear of being punished was found as a major factor for clinical incident reporting behavior of the health workers [25] and it is also evident that health care personnel are afraid of being punished if they report medical errors [26]. According to the Ethiopian penal code, it is punishing to injure a patient as a result of negligence. Therefore, the health workers may not disclose incidents due to fear of legal penalty.

Lack of feedback on clinical incident reporting was also a factor that affects the clinical incident reporting behavior of health workers. Health workers who perceive a lack of feedback on clinical incident reporting were 69.3% less likely to disclose incidents than those who did not so. Feedbacks from clinical risk management system may provide important information to staff on how to reduce and prevent incidents [27], and the structure and ideas through feedback were quite useful in developing a deeper comprehension of the events [28]. Hospital management should provide timed feedback and intervene in visible corrective measures on ongoing activities [29]. The importance of providing actionable feedback that improves systems is recognized as a key factor in encouraging future reporting with in the health workers [30].

Finally, lack of root cause analysis was also identified by the qualitative study as a factor for not reporting errors by the health workers. A similar result in a qualitative study was disclosed from a study conducted in Iran [24]. Identifying the cause of the incidents is important to prevent the errors through enables the creation of effective measures to reduce the likelihood of similar negative events occurring in the future [31, 32]. Likewise, root cause analysis is a process of investigating the root cause or sources of mistakes and vulnerabilities in a system to reduce the rate of adverse events [33].

### Limitation of the study

Social desirability bias could be the limitation of this study. Respondents were assured complete anonymity and confidentiality throughout the study, which is important to reduce social desirability bias. But still there might be a social desirability bias about behaviors of reporting clinical incidents. The study being conducted in a single institution could also be a limitation. As a result, it is recommended to conduct a mixed method study across the country to enhance the health-care system.

## Conclusions

This study showed that the overall clinical incident reporting behavior of the health professionals was very low. Training on clinical incident reporting, clinical incident reporting help to minimize errors, fear of legal penalties, lack of feedback, lack of organizational structure, and lack of investigation on the cause of errors were identified factors on clinical incident reporting behavior. Therefore, clinical incident reporting guidelines and a format for health care institutions should be established by Ethiopia’s minister of health, and health professionals should get training on clinical incident reporting. In addition, there should be also an investigation on the causes of medical errors.

## Supplementary Information

**Additional file 1.**

**Additional file 2.**

## Data Availability

Data will be available upon request from the corresponding author.

## Abbreviations

AOR: Adjusted odds ratio
SPSS: Statistical Product for Service Solution
