# Supplementary material for: Clinical incident reporting behaviors and associated factors among health professionals in Dessie comprehensive specialized hospital, Amhara Region, Ethiopia: a mixed method study
Source: BMC Health Serv Res. 2021 Dec 11;21:1331. doi: 10.1186/s12913-021-07350-y (PMC8666041; doi:10.1186/s12913-021-07350-y)
Supplement: Supplementary file 1 — Additional file 1. [file 12913_2021_7350_MOESM1_ESM.docx]

Table: Sociodemographic characteristics of the discussants on clinical incident reporting behavior in Dessie comprehensive specialized hospital, (n=18).

| **Participant Code** | **Sex** | **Age** | **Educational level** | **Profession** | **Working unit** | **Working Experience** | **Date of discussion** |
| --- | --- | --- | --- | --- | --- | --- | --- |
| P1F | M | 40 | BSc | Nursing | ART OPD | 7yrs | 21/03/2020 |
| P2F | F | 28 | BSc | Nursing | Pediatric | 6 yrs. | 21/03/2020 |
| P3F | M | 28 | General practitioner | Medicine | Surgery | 01 yrs. | 21/03/2020 |
| P4F | M | 34 | BSc | Anesthesia | OR | 6 yrs. | 21/03/2020 |
| P5F | F | 32 | BSc | Midwifery | Gynecology ward | 6 yrs. | 21/03/2020 |
| P6F | M | 27 | BSc | Pharmacy | Pedi pharmacy | 2 yrs. | 21/03/2020 |
| P1S | M | 32 | Specialist | Medicine | Gynecology ward | 3 yrs. | 23/03/2020 |
| P2S | F | 28 | BSc | Midwifery | Obstetric ward | 5 yrs. | 23/03/2020 |
| P3S | M | 34 | BSc | Nursing | Surgery | 2 yrs. | 23/03/2020 |
| P4S | F | 32 | BSc | Pharmacy | Central pharmacy | 4 yrs. | 23/03/2020 |
| P5S | M | 28 | BSc | Nursing | Emergency | 3 yrs. | 23/03/2020 |
| P6S | M | 28 | Diploma | Nursing | Medical | 2 yrs. | 23/03/2020 |
| P1T | F | 21 | BSc | Midwifery | Obstetric | 01 yrs. | 27/03/2020 |
| P2T | M | 29 | BSc | Anesthesia | OR | 01 yrs. | 27/03/2020 |
| P3T | M | 35 | BSc | Nursing | Medical ward | 6 yrs. | 27/03/2020 |
| P4T | M | 27 | BSc | Pediatric nursing | Pediatric | 4 yrs. | 27/03/2020 |
| P5T | M | 30 | BSc | Midwifery | Gynecology ward | 2 yrs. | 27/03/2020 |
| P6T | M | 29 | BSc | Pharmacy | Emergency pharmacy | 5 yrs. | 27/03/2020 |
