# Supplementary material for: Clinical incident reporting behaviors and associated factors among health professionals in Dessie comprehensive specialized hospital, Amhara Region, Ethiopia: a mixed method study
Source: BMC Health Serv Res. 2021 Dec 11;21:1331. doi: 10.1186/s12913-021-07350-y (PMC8666041; doi:10.1186/s12913-021-07350-y)
Supplement: Supplementary file 2 — Additional file 2. [file 12913_2021_7350_MOESM2_ESM.docx]

**ANNEX**

## Annex: Consent Form

Title of the study: **Clinical Incident Reporting Behaviors and Associated Factors among Health professionals in Dessie Comprehensive Specialized Hospital, Amhara Region, Ethiopia: A Mixed Method Study**.

Dear participant, my name is-----------------; and I am collecting data for research intended to assess clinical Incident Reporting Behaviors and Associated Factors among Health professionals.

Now, I want to ask you about your willingness to participate in this study. The study is important for this hospital to give better quality care and enhance patient safety culture with designing mutual and voluntary incident reporting. I would like to tell you as you have full rights to participate or not participate on this study and as you have right to stop participating at any time in the process of study. Additionally, all information you will give me are confidential and used only for this study.

Do you agree to participate?

A). yes

B). no

| No | **Question** |  |
| --- | --- | --- |
| 1. **Socio-demographic characteristics** | | |
| 101 | How old are you? | Age ____________(in years) |
| 102 | What is your sex? | 1. Male 2. Female |
| 103 | What is your marital status? | 1. Married  2. Single |
| 104 | What is your religion? | 1. Orthodox 2. Muslim 3. Protestant 4. Other (specify)_______ |
| 105 | What is your ethnicity? | 1. Amhara 2. Tigre 3. Oromo 4. Other (specify)____ |
| 106 | What is your department? | 1. Nursing 3. Midwifery  2. Anaesthesia 4. Medicine  5. Pharmacy |
| 107 | What is your level of education? | 1. 1. Specialist 2. 2. Medical doctor (1^st^ degree) 3. 3. MSc 4. 4. BSc 5. Diploma |
| 108 | Which ward/unit you currently work? | 1. Medicine 2. Surgery 3. Paediatrics 4. Gynaecology/Obstetrics 5. Others (specify ) _______________ |
| 109 | How many hours you work per week in this unit? | ______________ hours/week |
| 110 | How long you have served? | ________________ |
| **2. Organizational Factors** | | |
| 201 | Have you ever taken training about incident reporting? | 1. Yes 2. No |
| 202 | Is there any guideline (policy) to report incidents (mistakes) in your hospital? | 1. Yes 2. No 3. I don’t know |
| 203 | Is there any incident reporting format available at your work area? | 1. Yes 2. No 3. I don’t know |

**3. Self-perceived behaviour to incident reporting (multiple selection)**

| 301 | What is the benefit of incident (mistake) reporting? | 1. To get immediate help for patient 2. To learn from mistakes 3. To develop a system to minimize repetition of incident 4. It has no benefit 5. Others (specify) _______________ |
| --- | --- | --- |
| 302 | Reporting of mistakes would be easy if reports were made to | 1. Colleague 2. Senior health workers 3. Team leader/ ward head 4. Medical director 5. Other (specify) _______________ |
| 303 | In your opinion, what is the most important barrier of incident (mistake) reporting? | 1. Non-supportive environment (culture of shame & blame) 2. Loss of prestige among colleagues 3. Fear of legal or financial penalties 4. Fear of administrative suctions 5. Lack of feedback 6. Other (specify) ________________ |
| **4.Health workers ’ Incident reporting behaviour** | | |
| 401 | When a mistake is made, but is caught and corrected before affecting the patient, how often is this reported? | 1. Always 2. Most of the time 3. Sometimes 4. Rarely 5. Not reported at all |
| 402 | When a mistake is made but has no potential harm to the patient, how often is this reported? | 1. Always 2. Most of the time 3. Sometimes 4. Rarely 5. Not reported at all |
| 403 | When a mistake is made, that could harm the patient, but does not, how often is this reported? | 1. Always 2. Most of the time 3. Sometimes 4. Rarely 5. Not reported at all |

**Interviewer Guideline for Focused Group Discussion**

**1**. How do you see the incident reporting practice in this hospital?

**a.** Do you report events at the hospital when something goes wrong?

**2**. Do you remember an error which was not reported?

**3**. If so, why didn’t you report?

**a**. What keeps you from reporting clinical incidents?
